# Supplementary material for: Cryo-EM structures of the ABCA4 importer reveal mechanisms underlying substrate binding and Stargardt disease
Source: Nat Commun. 2021 Oct 8;12:5902. doi: 10.1038/s41467-021-26161-7 (PMC8501128; doi:10.1038/s41467-021-26161-7)
Supplement: Supplementary file 1 — Supplementary Information [file 41467_2021_26161_MOESM1_ESM.pdf]

SUPPLEMENTARY INFORMATION

Cryo-EM structures of the ABCA4 importer reveal mechanisms underlying substrate binding and Stargardt disease

Jessica Fernandes Scortecci, Laurie L. Molday, Susan B. Curtis, Fabian A. Garces, Pankaj Panwar, Filip Van Petegem, Robert S. Molday

Supplementary Material

Supplementary Table I:

Cryo-EM data collection, refinement and validation statistics

	**ABCA Apo** (EMDB-23617) (PDB 7M1P)	**ABCA4.N-Ret-PE complex** (EMDB-23618) (PDB 7M1Q)
**Data collection and processing**		
Magnification	81,000	81,000
Voltage (kV)	300	300
Electron exposure (e–/Å^2)	50	50
Defocus range (μm)	-0.8 / -2.2	-0.8 / -2.2
Pixel size (Å)	0.5295	0.5295
Symmetry imposed	C1	C1
Initial particle images (no.)	2,166,875	2,699,426
Final particle images (no.)	100,459	209,790
Map resolution (Å)	3.6	2.92
FSC threshold	0.143	0.143
Map resolution range (Å)	3.0-4.8	2.6-4.4
**Refinement**		
Initial model used (PDB code)	5XJY	
Model resolution (Å)	3.6	2.92
FSC threshold	0.143	0.143

Model resolution range (Å)		
Map sharpening *B* factor ($Å^2$)	Local sharpening	-
Model composition		
Non-hydrogen atoms	13517	13783
Protein residues	1938	1912
Ligands	13	15
B factors ($Å^2$)		
Protein	59.02	60.06
Ligand	69.73	75.75
R.m.s. deviations		
Bond lengths (Å)	0.002	0.003
Bond angles (°)	0.562	0.618
Validation		
MolProbity score	1.97	1.90
Clashscore	7.97	8.09
Poor rotamers (%)	0.0	0.08
Ramachandran plot		
Favored (%)	90.47	92.67
Allowed (%)	9.48	7.33
Disallowed (%)	0.05	0.0

Supplementary Table 2: Primers used to construct ABCA4 Variants

ABCA4 Variant	Primer Sequence
ABCA4-W339G	F: 5'CTC CTT CAA CGG GTA TGA AGA CAA TAA CTA TAA GGC-3'<br>R: 5'AGC ACC CGA GAG CCA CCT-3'
ABCA4-R653C	F: 5' GAT CAT CCT GAA CTG CTG TTT CCC TAT CTT CAT GG-3'<br>R: 5' AGA TAG GGA AAC AGC AGT TCA GGA TGA TCA TG-3'
ABCA4-R587A	F: 5' GAT TAA AGA CGC GTA TTG GGA TTC TGG TCC-3'<br>R: 5' CCC AAT ACG CGT CTT TAA TCT TAT TGG-3'
ABCA4-Y345C	F: 5' GAC AAT AAC TGT AAG GCC TTT CTG GG-3'<br>R: 5'GGC CTT ACA GTT ATT GTC TTC ATA CCA G-3'
ABCA4-Y345A	F: 5'GAC AAT AAC GCT AAG GCC TTT CTG GGG ATT G-3'<br>R: 5' GGCCTTAGCGTTATTGTCTTCATACCAG-3'

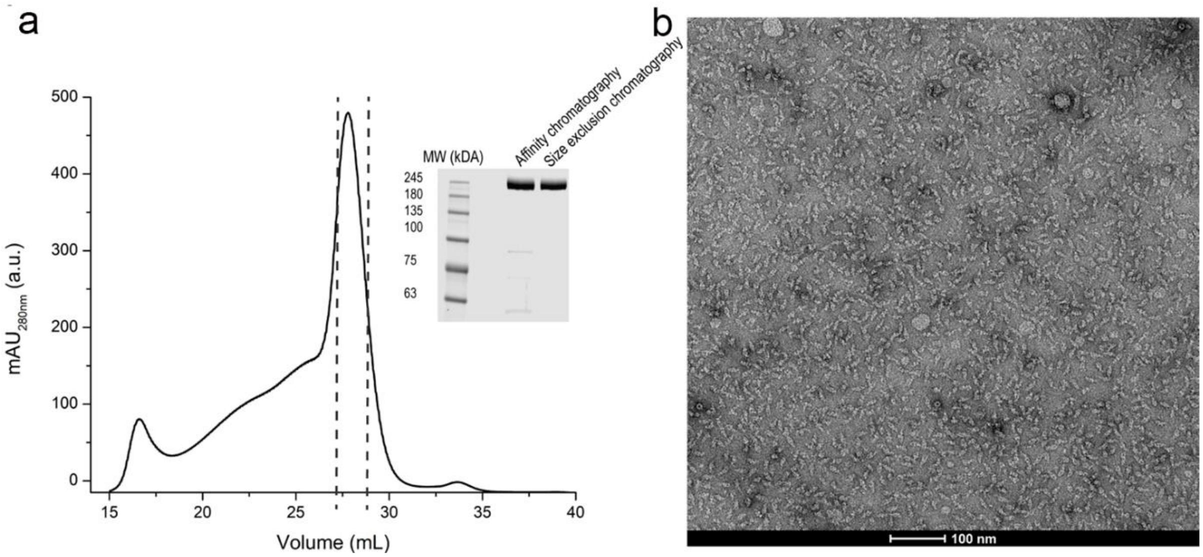

Supplementary Figure 1. Purification and characterization of ABCA4. **a** Chromatogram from size exclusion chromatography (SEC) using a Superose 6 column. Dashed lines: fraction collected for EM analysis. Inset: Coomassie blue stained 8% SDS-gel from the 1D4 affinity purification step and subsequent SEC indicating a high degree of purity in the final SEC step. The chromatogram and SDS-PAGE are a representative of > 12 independent experiments that showed similar results. **b** Negative stained (NS) EM image of purified ABCA4 at 49,000X magnification. The micrograph is a representative of 30 NS-EM images

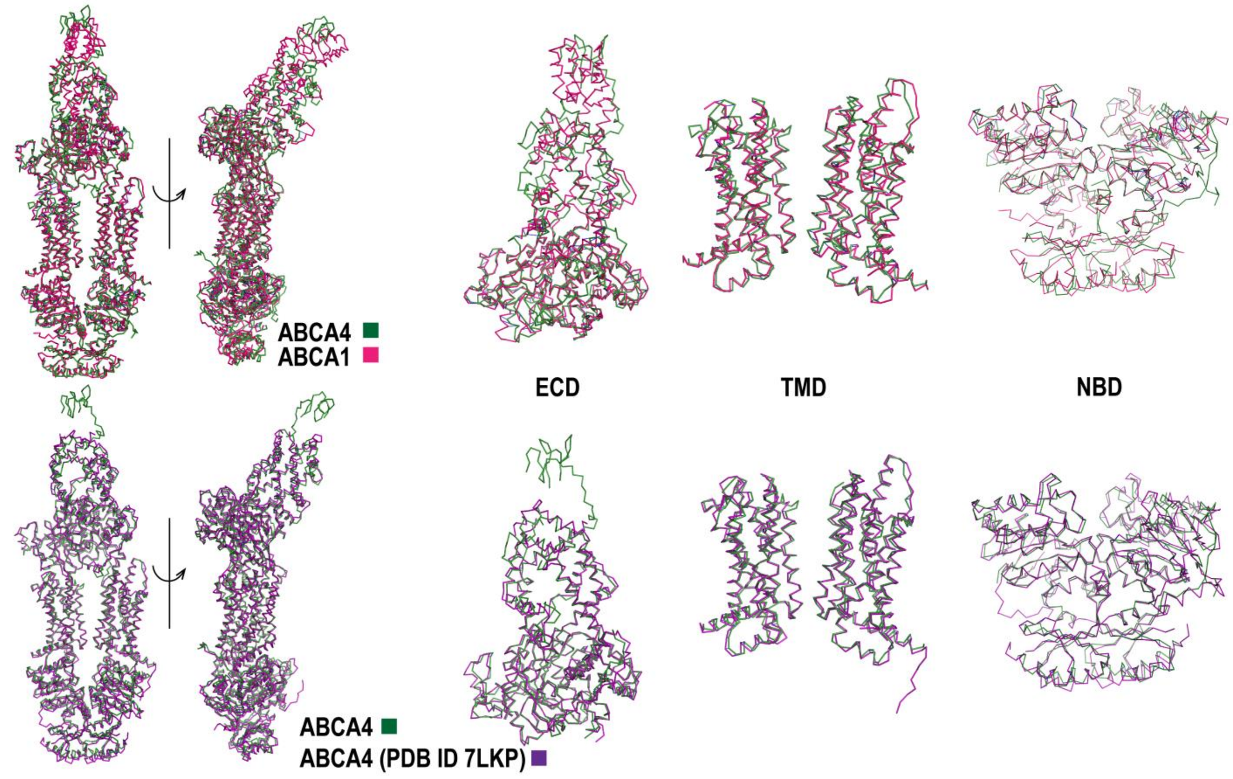

Supplementary Figure 2. Superposition of ABCA4 (PDB ID 7M1P – green) with ABCA1 (PDB ID 5XJY – pink) and ABCA4 (PDB ID 7LKP – purple). Protein is represented as ribbons and the alignment of each domain is also shown. ABCA4 and ABCA1 show the canonical ABCA folding with each domain being slightly different with Cα RMSD = 1.6 Å for 400 atoms in the transmembrane domains (TMDs), Cα RMSD = 2.0 Å for 496 atoms in the exocytoplasmic domains (ECDs) and Cα RMSD = 2.2 Å for 343 atoms in the nucleotide binding domains (NBDs). The superposition of both ABCA4 structures (PBD ID 7M1P and PBD ID 7LKP) indicates that they are remarkably similar, with Cα RMSD = 0.9 Å for TMDs (485 atoms), Cα RMSD = 0.8 Å for ECDs (550 atoms) and Cα RMSD = 1.0 Å for NBDs (465 atoms).

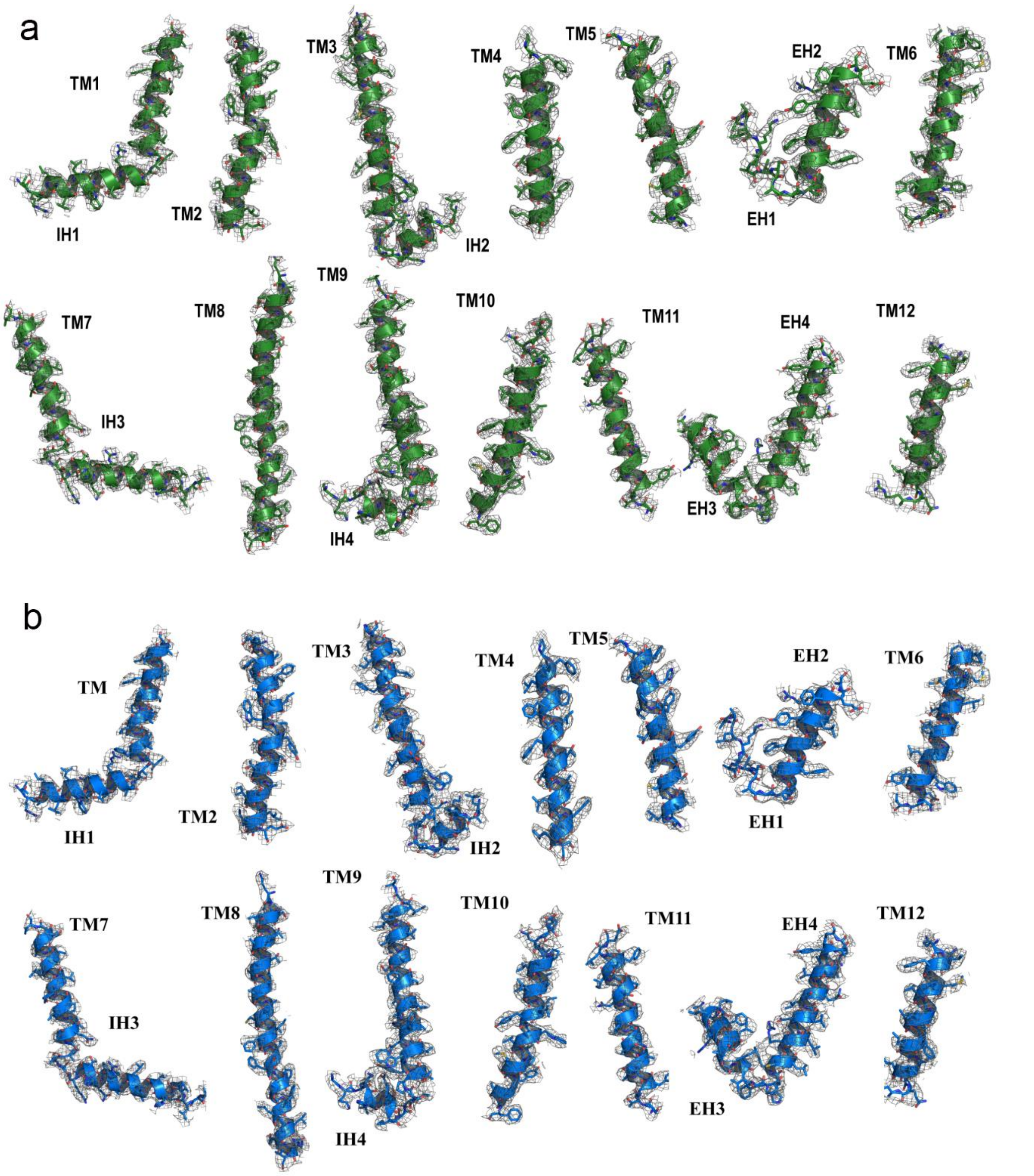

Supplementary Figure 3. **Electron microscope (EM) densities for helices in the transmembrane domains (TMD). a** ABCA4 in unbound state and **b** ABCA4 in the substrate complex state. The EM densities are shown as grey mesh with σ = 6.0

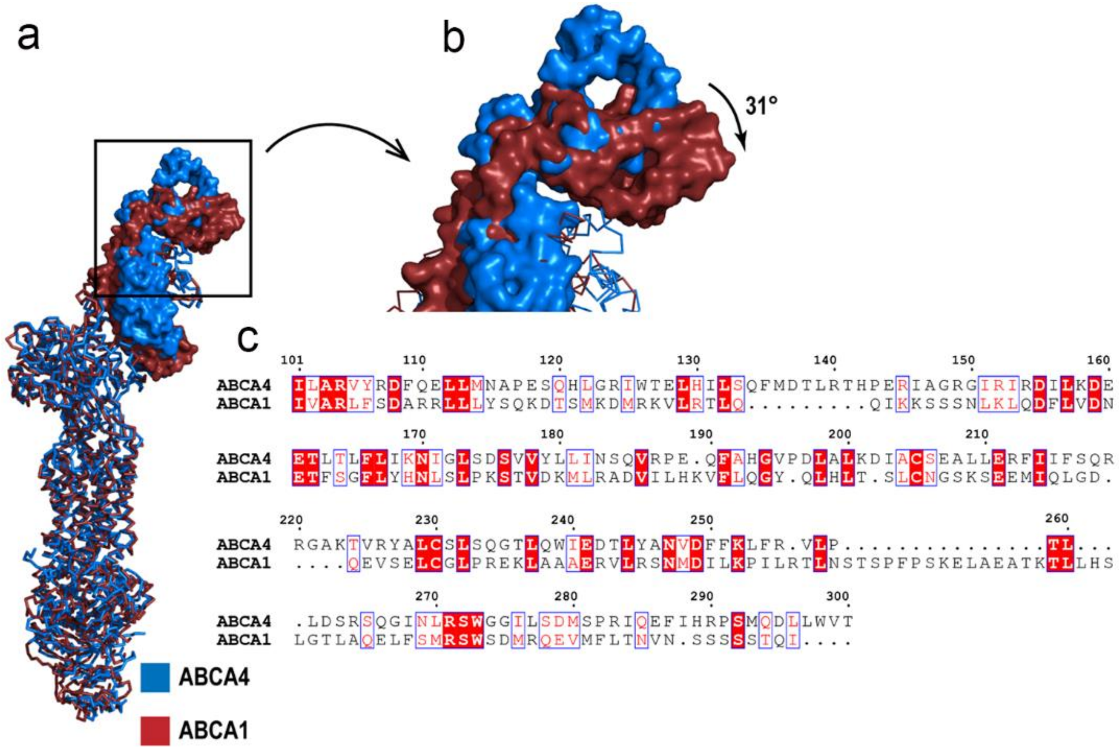

Supplementary Figure 4. Comparison of the exocytoplasmic domain (ECD) of ABCA4 (blue) and ABCA1 (red) (PDB ID 5XJY). **a** Surface representation for the lid portion of ECD. Protein is represented as ribbon. **b** A closer view of the lid region indicates a tilt of around 31º between both proteins. **(c)** Alignment of the lid region shows that they share only 24.2% sequence identity.

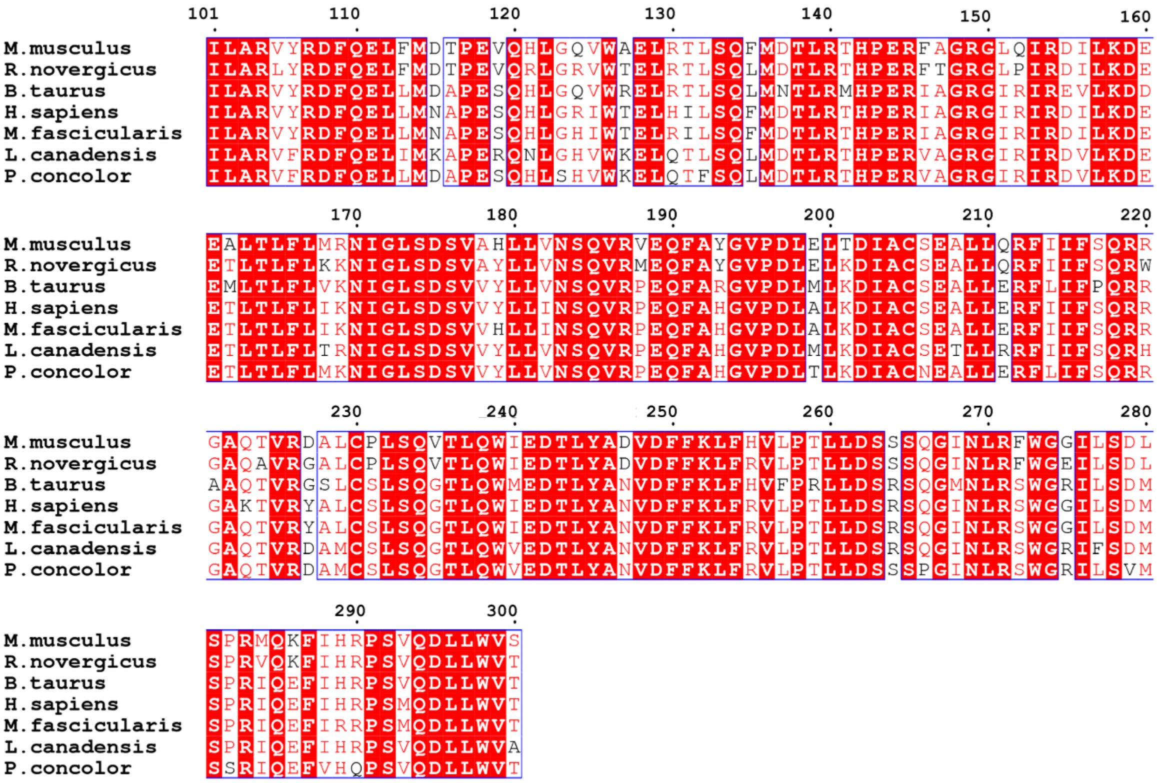

Supplementary Figure 5. Sequence alignment of the lid portion from extracellular domain. Alignment of amino acid 100 to 300 of ABCA4 from various species indicates that this region is highly conserved.

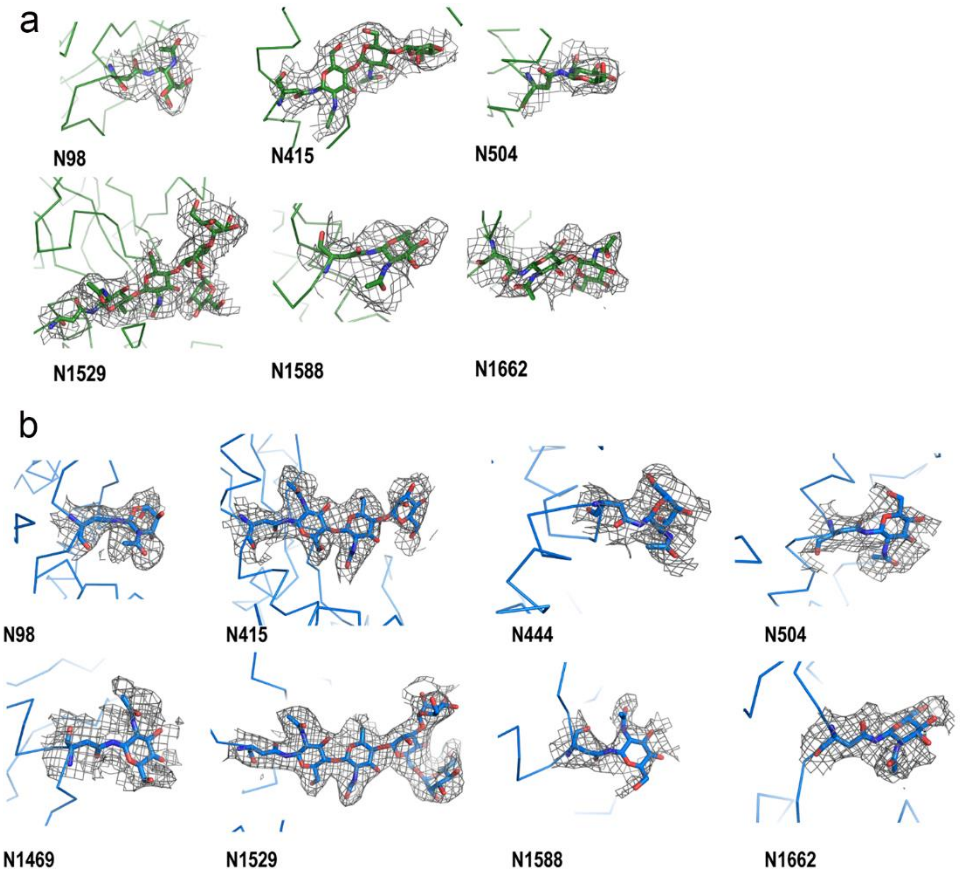

Supplementary Figure 6. **EM densities for N-linked glycosylation sites**. **a** ABCA4 in unbound state and **b** ABCA4 in the complex state. The EM densities are shown as grey mesh with σ = 5.0

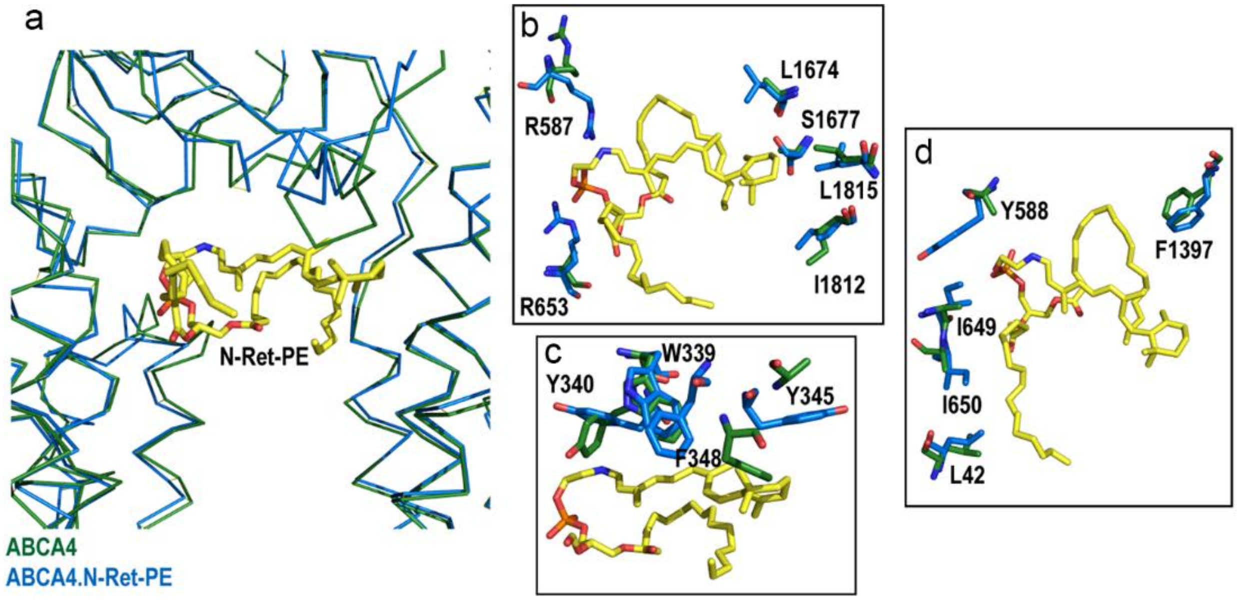

Supplementary Figure 7. **Superposition of substrate-free state (green) and substrate-bound state (blue). a** Ribbon representation indicates no significant conformational changes between both states (RMSDs: TMD1= 0.505 Å, TMD2 = 0.482 Å, ECD1 = 0.474 Å). **b-d** Residues involved in substrate pocket present a slightly different side chain orientation, indicating the residues accommodate locally to allow substrate binding.

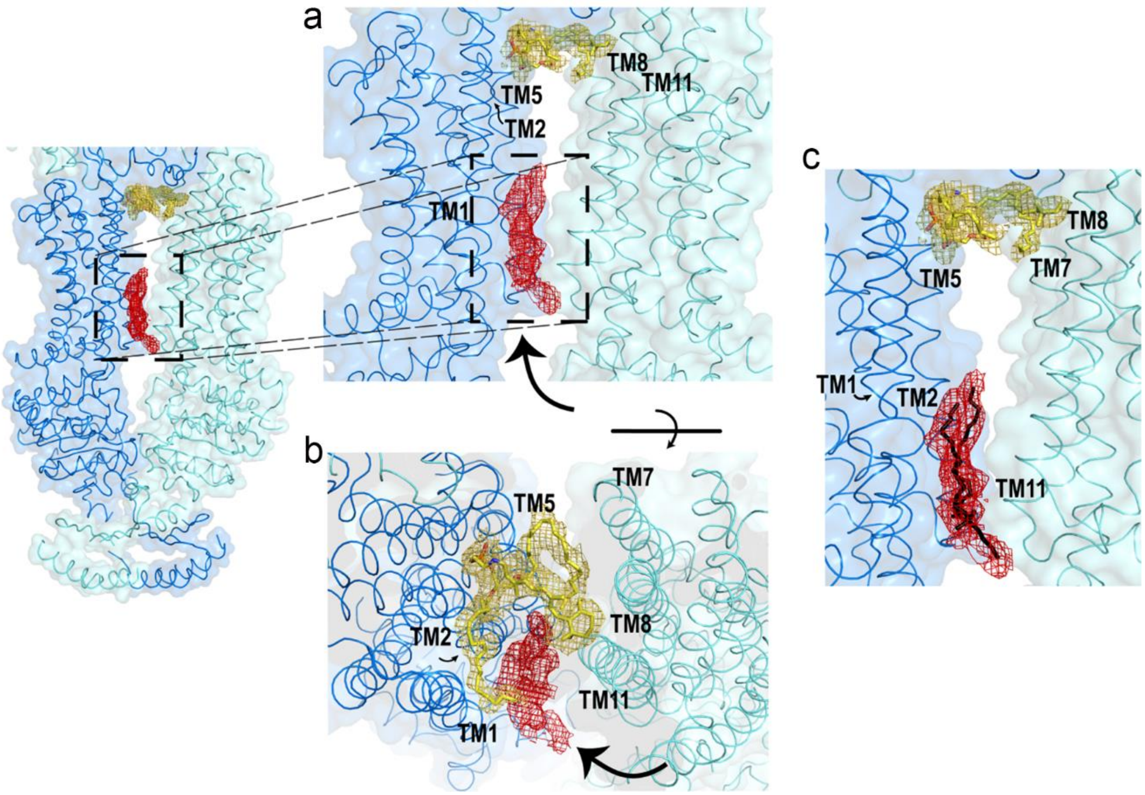

Supplementary Figure 8. Surface and ribbon representations of ABCA4-N-Ret-PE complex. **a** EM density, with σ = 6.0, that resembles a lipid (red) is located in the same position in the unbound state, indicated with an arrow, implicating it as a structural lipid. **b** Orthogonal view. **c** The most probable orientation of the lipid (black) between TMDs. The EM density for N-Ret-PE is also shown, with σ = 6.0. The N- and C- halves are colored as blue and light blue, respectively.

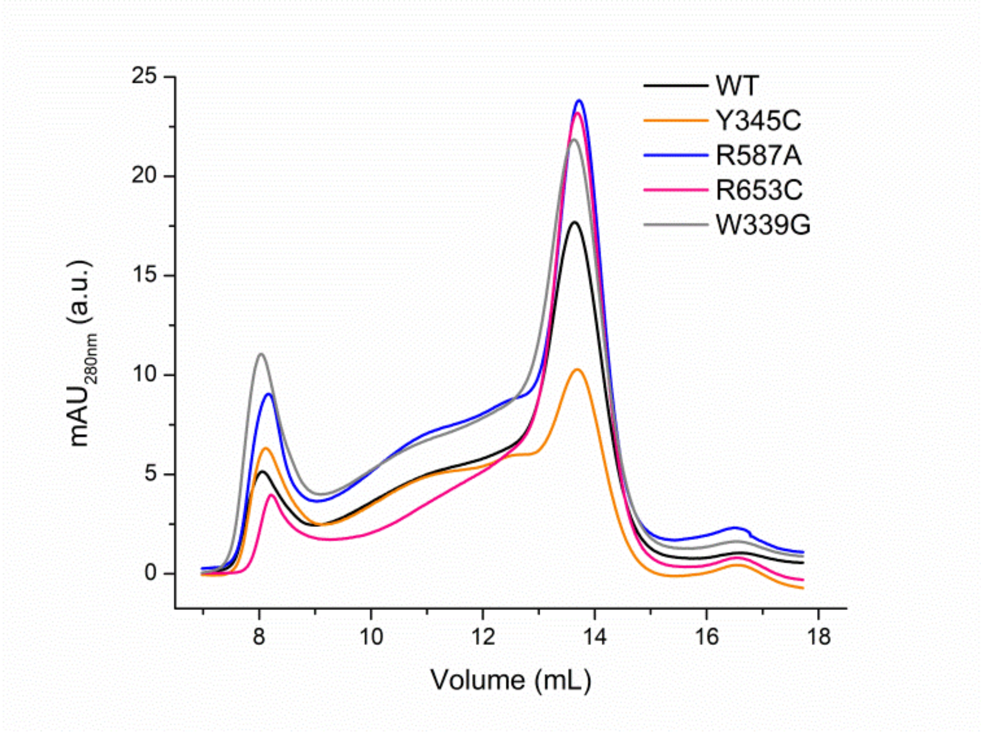

Supplementary Figure 9. Size exclusion profiles for the ABCA4 variants involving residues within the substrate binding pocket. The chromatograms obtained for the ABCA4 variants display the same profile as WT, suggesting that these mutations do not significantly affect protein folding. The Y345C variant, however, shows a reduced proportion of the major monomer peak.

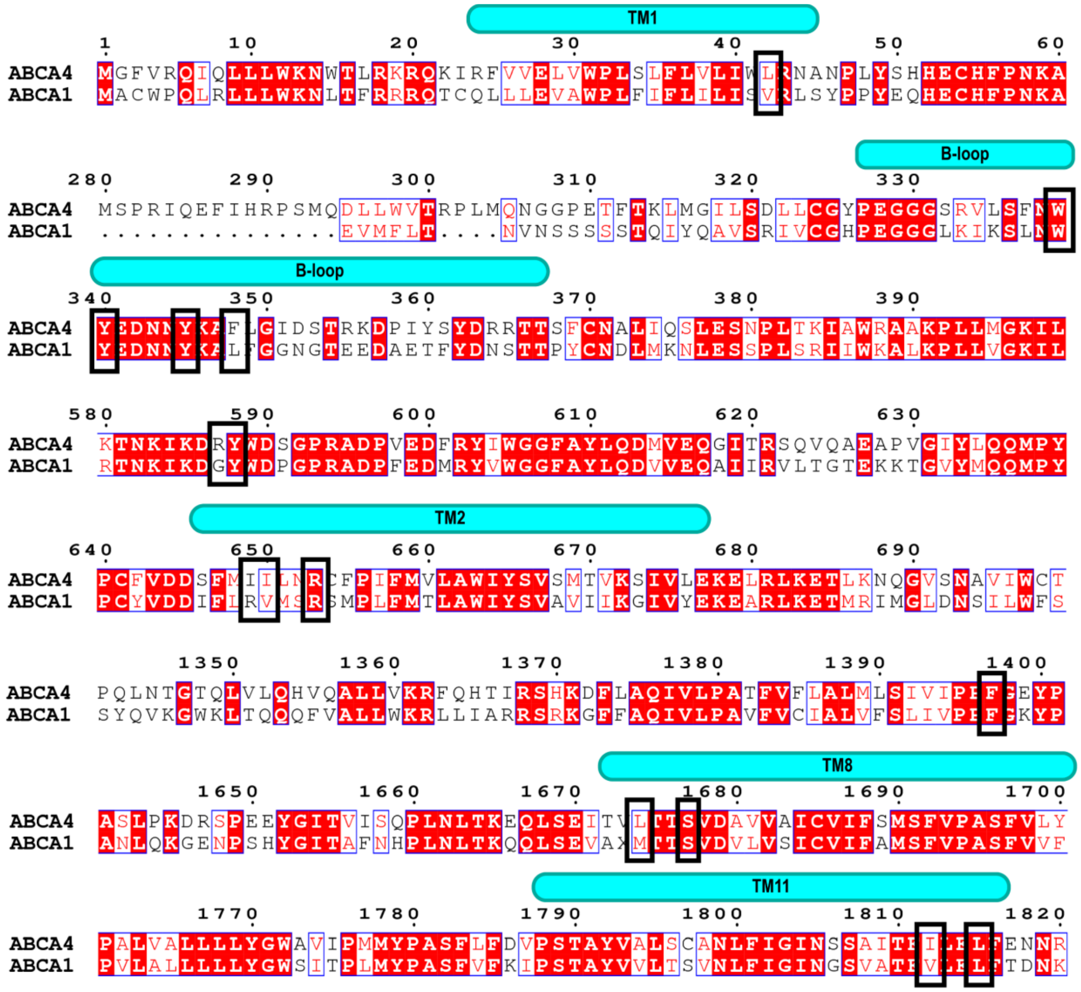

Supplementary Figure 10. Sequence alignment between ABCA4 and ABCA1 for the regions within the binding pocket. The residues involved in the substrate binding of ABCA4 are highlighted with a black box.

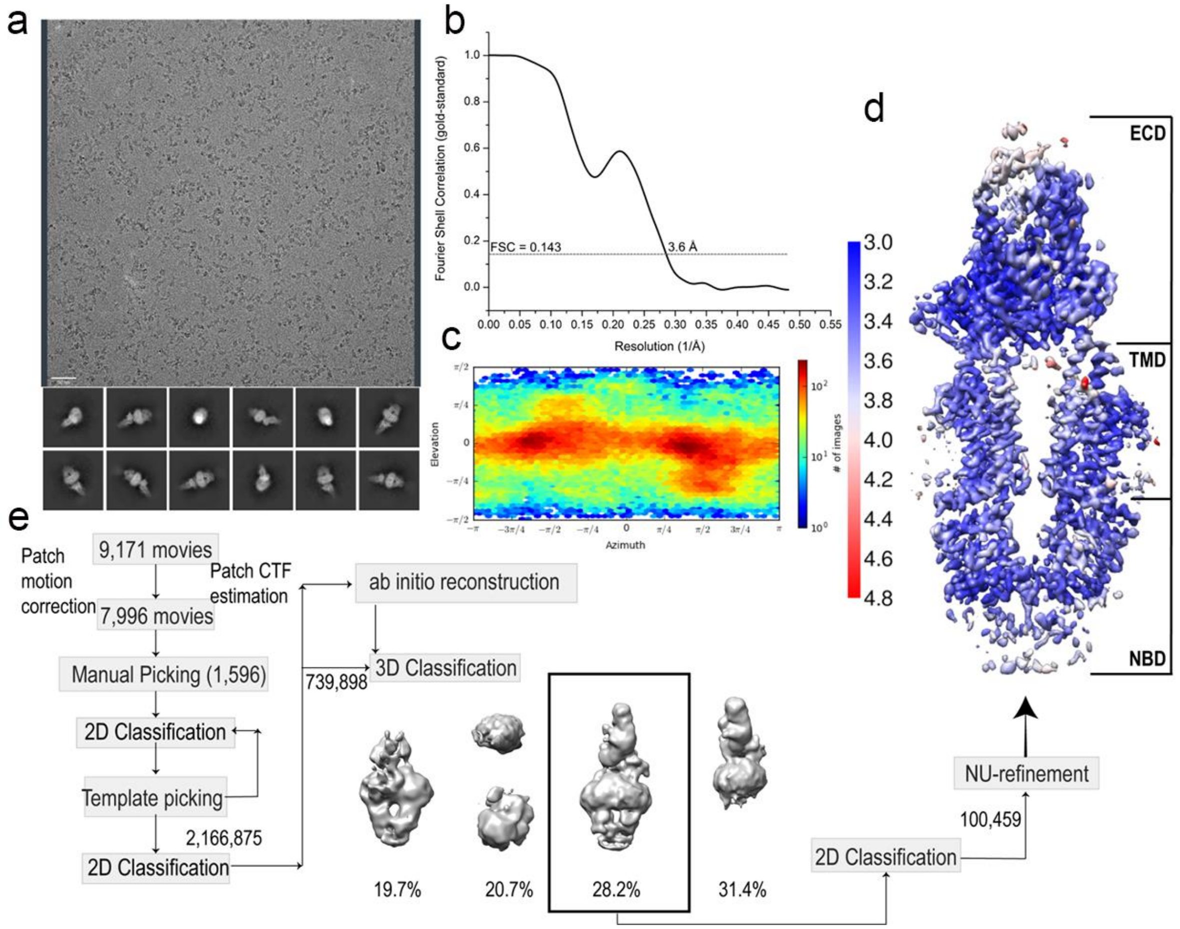

Supplementary Figure 11. Cryo-EM of ABCA4 in the unbound state. **a** Representative cryo-EM micrograph and 2D class average. Bar: 50 nm. The micrograph is a representative of 9,171 cryo-EM images. **b** Gold standard Fourier Shell Correlation (FSC) indicates a final resolution of 3.6 Å. **c** Azimuth plot of angular distribution does not show preferred orientation. **d** Local resolution map for ABCA4 in its apo state calculated in cryoSPARC v.3.0 **e** Workflow for data processing using cryoSPARC v3.0.

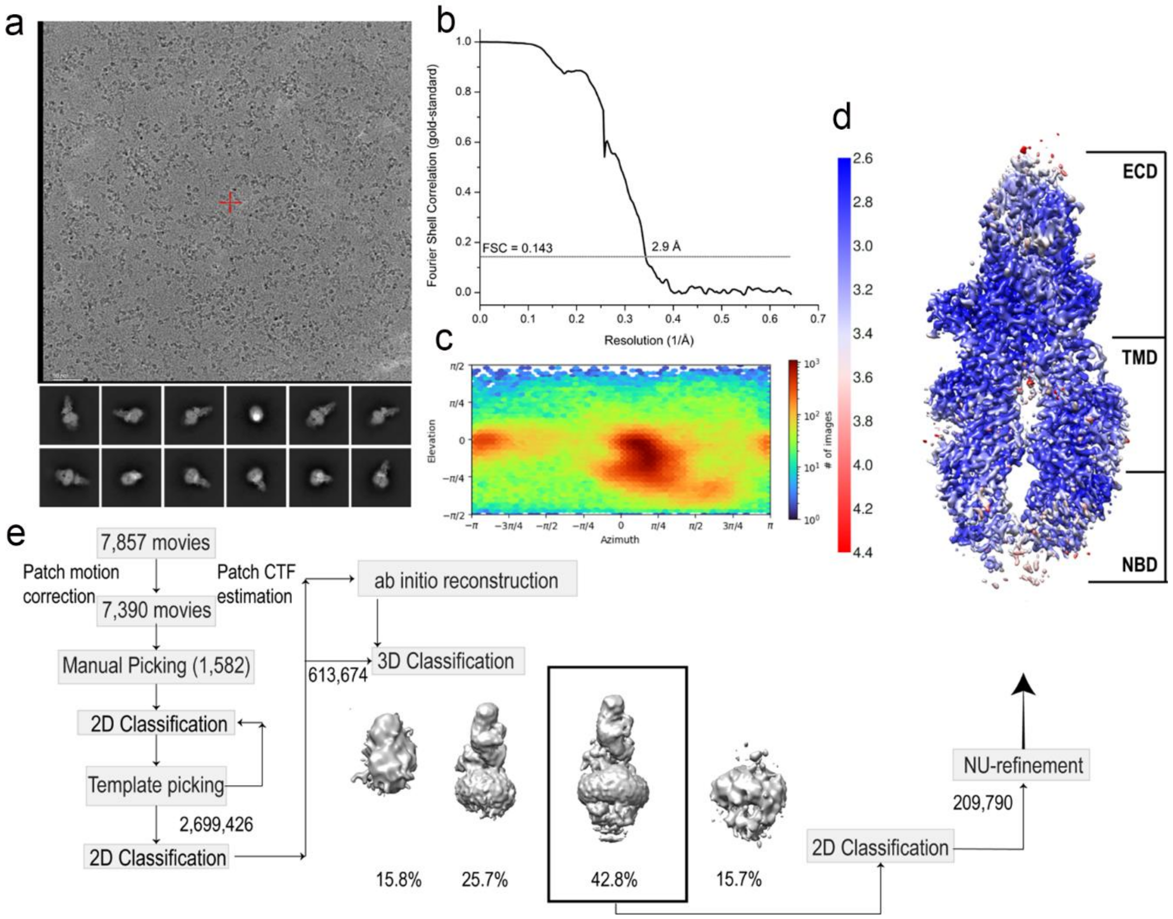

Supplementary Figure 12. Cryo-EM of ABCA4 in complex with N-Ret-PE. **a** Representative cryo-EM micrograph and 2D class average. Bar: 50nm. The micrograph is a representative of 7,857 cryo-EM images **b** Gold standard Fourier Shell Correlation (FSC) indicates a final resolution of 2.9 Å. **c** Azimuth plot of angular distribution does not show preferred orientation. **d** Local resolution map for ABCA4 complexed with N-Ret-PE calculated in cryoSPARC v.3.0 **e** Data processing workflow using cryoSPARC v3.0.

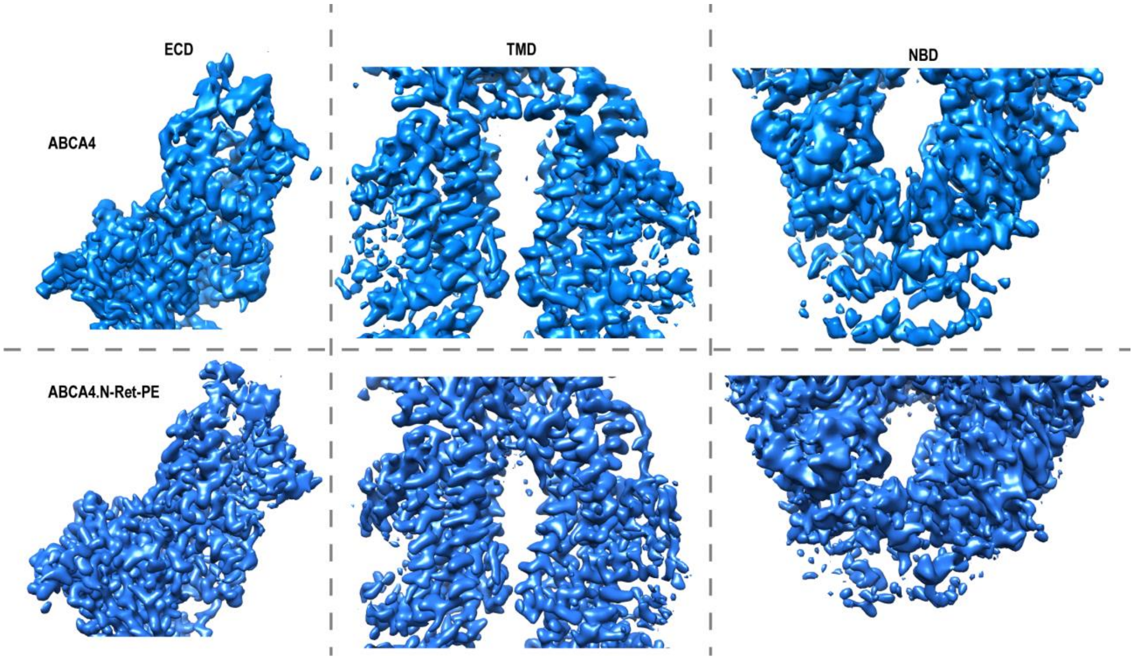

Supplementary Figure 13. Cryo-EM maps focusing on each domain. Unbound state (upper panel) and in complex with N-Ret-PE (lower panel).
