# Supplementary material for: Cryo-EM structures of the ABCA4 importer reveal mechanisms underlying substrate binding and Stargardt disease
Source: Nat Commun. 2021 Oct 8;12:5902. doi: 10.1038/s41467-021-26161-7 (PMC8501128; doi:10.1038/s41467-021-26161-7)
Supplement: Supplementary file 3 — Reporting Summary [file 41467_2021_26161_MOESM3_ESM.pdf]

nature research

Corresponding author(s): Robert S. Molday

Last updated by author(s): 9/1//21

Reporting Summary

Nature Research wishes to improve the reproducibility of the work that we publish. This form provides structure for consistency and transparency in reporting. For further information on Nature Research policies, see our Editorial Policies and the Editorial Policy Checklist.

Statistics

For all statistical analyses, confirm that the following items are present in the figure legend, table legend, main text, or Methods section.

n/a	Confirmed	
☐	☒	The exact sample size (*n*) for each experimental group/condition, given as a discrete number and unit of measurement
☐	☒	A statement on whether measurements were taken from distinct samples or whether the same sample was measured repeatedly
☐	☒	The statistical test(s) used AND whether they are one- or two-sided<br>*Only common tests should be described solely by name; describe more complex techniques in the Methods section.*
☒	☐	A description of all covariates tested
☐	☒	A description of any assumptions or corrections, such as tests of normality and adjustment for multiple comparisons
☐	☒	A full description of the statistical parameters including central tendency (e.g. means) or other basic estimates (e.g. regression coefficient) AND variation (e.g. standard deviation) or associated estimates of uncertainty (e.g. confidence intervals)
☐	☒	For null hypothesis testing, the test statistic (e.g. *F*, *t*, *r*) with confidence intervals, effect sizes, degrees of freedom and *P* value noted<br>*Give P values as exact values whenever suitable.*
☒	☐	For Bayesian analysis, information on the choice of priors and Markov chain Monte Carlo settings
☒	☐	For hierarchical and complex designs, identification of the appropriate level for tests and full reporting of outcomes
☒	☐	Estimates of effect sizes (e.g. Cohen's *d*, Pearson's *r*), indicating how they were calculated

Our web collection on statistics for biologists contains articles on many of the points above.

Software and code

Policy information about availability of computer code

Data collection	CryoEM data was collected with SerialEM v3.7
Data analysis	CryoSPARC v.3.0; Modeller v.9.25; Chimera v1.14, Cootv.0.9; Phenix v.1.19-4092; PyMOL 1.7; GraphPad Prism 9.0

For manuscripts utilizing custom algorithms or software that are central to the research but not yet described in published literature, software must be made available to editors and reviewers. We strongly encourage code deposition in a community repository (e.g. GitHub). See the Nature Research guidelines for submitting code & software for further information.

Data

Policy information about availability of data

All manuscripts must include a data availability statement. This statement should provide the following information, where applicable:

- Accession codes, unique identifiers, or web links for publicly available datasets
- A list of figures that have associated raw data
- A description of any restrictions on data availability

The cryo-EM density maps have been deposited in the Electron Microscopy Data Bank under accession codes: EMD-23617 for substrate-free ABCA4 and EMD-23618 for substrate-bound ABCA4 and the coordinates have been deposited in the Protein Data Bank under the accession codes: PDB ID 7M1P for substrate-free ABCA4 and PDB ID 7M1Q for substrate-bound ABCA4. Source data are provided as supplementary information with this paper.

Field-specific reporting

Please select the one below that is the best fit for your research. If you are not sure, read the appropriate sections before making your selection.

☒ Life sciences ☐ Behavioural & social sciences ☐ Ecological, evolutionary & environmental sciences

For a reference copy of the document with all sections, see nature.com/documents/nr-reporting-summary-flat.pdf

Life sciences study design

All studies must disclose on these points even when the disclosure is negative.

Sample size	Sample sizes obtained from cryoEM structure, also in vitro analysis, are in agreement with the literature. Previous studies have shown that these sample sizes are sufficient
Data exclusions	No data was excluded
Replication	3 replicate measurements for each experiment were performed. Three or more independent experiments were performed unless indicated otherwise.
Randomization	Not relevant to this study. CryoEM data collection is not a random process
Blinding	Not relevant as there was no grouping of the samples

Reporting for specific materials, systems and methods

We require information from authors about some types of materials, experimental systems and methods used in many studies. Here, indicate whether each material, system or method listed is relevant to your study. If you are not sure if a list item applies to your research, read the appropriate section before selecting a response.

Materials & experimental systems

n/a	Involved in the study	
☐	☒	Antibodies
☐	☒	Eukaryotic cell lines
☒	☐	Palaeontology and archaeology
☒	☐	Animals and other organisms
☒	☐	Human research participants
☒	☐	Clinical data
☒	☐	Dual use research of concern

Methods

n/a	Involved in the study	
☒	☐	ChIP-seq
☒	☐	Flow cytometry
☒	☐	MRI-based neuroimaging

Antibodies

Antibodies used	Rho 1D4 antibody was produced in our lab in 1982 and has been widely used in the research community. Available from multiple commercial sources including Millipore-Sigma as well as from UBC for large quantities used in protein purification
Validation	Specificity of this antibody against the 1D4 epitope has been validated using ABCA4 vs ABCA4-1D4 in cell expression studies and western blotting.

Eukaryotic cell lines

Policy information about cell lines

Cell line source(s)	HEK293T and HEK293F cells were purchased from ATCC and Invitrogen and authenticated by these companies.
Authentication	HEK293T cells were obtained from commercial source with validation documentation and were only used for transfection and protein expression purposes.
Mycoplasma contamination	No contamination
Commonly misidentified lines (See ICLAC register)	No commonly misidentified cell lines were used in this study.
